# CD180 Ligation Inhibits TLR7- and TLR9-Mediated Activation of Macrophages and Dendritic Cells Through the Lyn-SHP-1/2 Axis in Murine Lupus

**DOI:** 10.3389/fimmu.2018.02643

**Published:** 2018-11-15

**Authors:** Yonghong Yang, Cuiling Wang, Panpan Cheng, Xiaobei Zhang, Xuehui Li, Yuan Hu, Feihong Xu, Feng Hong, Guanjun Dong, Huabao Xiong

**Affiliations:** ^1^Institute of Immunology and Molecular Medicine, Jining Medical University, Jining, China; ^2^Department of Central Laboratory, Affiliated Hospital of Jining Medical University, Jining, China; ^3^Department of Hematology, Affiliated Hospital of Jining Medical University, Jining, China; ^4^Department of Medicine, Immunology Institute, Icahn School of Medicine at Mount Sinai, New York, NY, United States

**Keywords:** CD180, TLR, SLE, macrophage, DCs

## Abstract

Activation of TLR7 and TLR9 by endogenous RNA- or DNA-containing ligands, respectively, can lead to hyper-activation of immune cells, including macrophages and DCs, subsequently contributes to the pathogenesis of SLE. CD180, a TLR-like protein, is specifically involved in the development and activation of immune cells. Our previous study and others have reported that CD180-negative B cells are dramatically increased in SLE patients and responsible for the production of auto-antibodies. However, the mode of CD180 expression on macrophages and DCs in SLE remains unclear and the role of CD180 on regulating TLR7- and TLR9-mediated activation of macrophages and DCs are largely unknown. In the present study, we found that the percentages of CD180-negative macrophages and DCs were both increased in SLE patients and lupus-prone MRL/*lpr* mice compared with healthy donors and wild-type mice, respectively. Notably, ligation of CD180 significantly inhibited the activation of TLR7 and TLR9 signaling pathways in macrophages and DCs through the Lyn-SHP-1/2 axis. What's more, injection of anti-CD180 Ab could markedly ameliorate the lupus-symptoms of imiquimod-treated mice and lupus-prone MRL/*lpr* mice through inhibiting the activation of macrophages and DCs. Collectively, our results highlight a critical role of CD180 in regulating TLR7- and TLR9-mediated activation of macrophages and DCs, hinting that CD180 can be regarded as a potential therapeutic target for SLE treatment.

## Introduction

Systemic lupus erythematosus (SLE) is an autoimmune disease characterized by a loss of immunologic tolerance and hyper-activation of immune cells, which lead to aberrant production of pro-inflammatory cytokines, generation of antinuclear antibodies and multi-organ damage (1). Numerous studies suggest that many types of immune cells, including macrophages and dendritic cells (DCs), exhibit hyper-activated phenotypes and functions in SLE (2–4). Abnormal activations of macrophages and DCs have been shown to contribute to the pathogenesis of SLE: inappropriate or dysfunctional antigen presentation by DCs might promote the breakdown of immunologic tolerance in SLE (5–7); monocytes/macrophages can infiltrate in target organ and contribute to SLE pathogenesis through the pro-inflammatory cytokines production (8–10). Thus, effectively and tightly control the hyper-activations of macrophages and DCs are critical for appropriate treatment of SLE.

Toll-like receptors (TLRs) are type I transmembrane proteins and located on various cellular membranes. Notably, TLR7 and TLR9, which recognize single-stranded RNA-associated and unmethylated CpG DNA-associated auto-antigens, respectively, are both implicated in the activation of immune cells and the initiation and development of SLE (11–13). It has been shown that the expressions of TLR7 and TLR9 are increased in SLE patients and highly correlated with the disease activity index (11, 14). Moreover, TLR7 and TLR9 polymorphisms are associated with the disease incidence of SLE (15–17). Abnormal regulations of TLR7 and TLR9 pathways are indeed critically involved in the activation of macrophages and DCs, subsequently break self-tolerance and contribute to the pathogenesis of SLE through antigen presentation and cytokines production, such as interferons, tumor necrosis factors (TNFs) and specific interleukins (18–20). Thus, TLR7 and TLR9 pathways have been regarded as important targets for therapeutic intervention of SLE. Many molecules have been shown to act as co-receptors and/or accessory molecules to regulate the activation of TLR7 and TLR9 pathways by using different mechanisms such as dissociation of adaptor complexes, degradation of signal proteins or transcriptional regulation (21, 22). For example, src homology region 2 domain-containing phosphatase-1 (SHP-1) and SHP-2, which are members of the protein tyrosine phosphatase (PTP) family, have been recently recognized as negative regulators of TLRs pathways (23–25). However, the identification of the negative regulators and details of the mechanisms by which TLR7 and TLR9 pathways are tightly controlled remain to be fully elucidated.

CD180, also known as RP105, is a 105 kD TLR-associated molecule that mainly expressed on macrophages, DCs and B cells (26). CD180 can influence the development, homeostasis and survival of immune cells, including macrophages, DCs and B cells, through phosphorylating various kinases, such as Lyn (27–31). Accumulating evidence has shown that CD180 is tightly associated with the pathogenesis of autoimmune diseases: CD180 can modulate the functions of antigen-presenting cells and regulate the development of collagen-induced arthritis; the population of CD180-negative B cell significantly increases in SLE patients and it changes in parallel with SLE disease activity (32, 33). Notably, a close relationship exists between CD180 and the activation of TLRs pathways already. CD180 enhances the activation of TLR4 pathway in B cells (34), but functions as an endogenous inhibitor of TLR4 pathway in other types of cells, including macrophages and DCs (35, 36). What's more, CD180 has been reported to enhance TLR2-mediated activation of macrophages (37). However, the mode of CD180 expression on macrophages and DCs in SLE remains unclear and the role of CD180 on regulating TLR7- and TLR9-mediated activation of macrophages and DCs are largely unknown.

In the present study, we found that the percentages of CD180-negative macrophages and DCs were both increased in SLE patients and lupus-prone MRL/*lpr* mice compared with healthy donors and wild-type mice, respectively. Notably, ligation of CD180 significantly inhibited TLR7- and TLR9-mediated activation of macrophages and DCs through the Lyn-SHP-1/2 axis. What's more, treatment of anti-CD180 Ab efficiently ameliorated the lupus-symptoms of lupus-prone mice. Taken together, these results suggest that CD180 plays a critical role in regulating the activation of TLR7 and TLR9 pathways in macrophages and DCs, hinting that CD180 may be used as a potential therapeutic target for SLE treatment.

## Materials and methods

### Mice

C57BL/6 mice (6- to 8-week old) were obtained from Pengyue Experimental Animal Breeding Company (China) and MRL/*lpr* mice (8-week old) were obtained from the Nanjing University Model Animal Research Centre. All the mice were maintained under specific pathogen-free conditions at Jining Medical University. Female mice used to generate BM-derived macrophages (BMDMs) and BM-derived macrophages (BMDCs) were 8- to 10-week old. To construct imiquimod-induced lupus-prone mice (IMQ-mice), the skin on the right ears of the mice were treated topically, 3 times weekly, with either 1.25 mg of 5% imiquimod cream (Med-Shine Pharmaceutical, China) from the 8th week to the 18th week. Under certain conditions, IMQ-induced lupus-prone mice received weekly intraperitoneal injections with 100 μg of anti-CD180 Ab (Biolegend) from the 8th week to the 18th week; MRL/*lpr* mice received weekly intraperitoneal injections with 100 μg of anti-CD180 Ab (Biolegend) from the 14th week to the 18th week. Rat IgG2b (Biolegend) was used as a control. All the mouse experiments were conducted in accordance with institutional guidelines for animal care and used based on the Guide for the Animal Care Committee at Jining Medical University.

### Antibodies

The following antibodies were used for immunoblotting: Cell Signaling Technology, anti-p38, anti-p-p38, anti-p65, anti-p-p65, anti-JNK, anti-p-JNK, anti-ERK, anti-p-ERK, anti-SHP-1, anti-p-SHP-1, anti-SHP-2, anti-p-SHP-2, anti-Lyn, anti-p-Lyn, the above antibodies were used at a 1:1,000 dilution; Beyotime Institute of Biotechnology, anti-β-actin (diluted at 1:1,000); HRP-labeled Goat anti-rabbit (1:3,000), HRP-labeled Rabbit anti-mouse (1:3,000). The following antibodies were used for flow cytometry: anti-human HLA-DR, anti-human CD14, anti-human CD11c, anti-human CD180, anti-human CD86, anti-mouse F4/80, anti-mouse CD11c, anti-mouse B220, anti-mouse CD40, anti-mouse CD86, anti-mouse CD180. All the antibodies used for flow cytometry were purchased from Biolegend and used at a 1:100 dilution. An isotype control was used for each antibody.

### Isolation of human peripheral blood mononuclear cells (PBMCs)

Whole blood samples were collected from SLE patients (*n* = 19, female) and healthy donors (*n* = 19, female). PBMCs were separated from plasma by Ficoll centrifugation (Lymphoprep, Nycomed, Oslo, Norway) according to the standard procedures and then the harvested PBMCs were used for subsequent experiments. All patients with SLE fulfilled the revised disease criteria of the American College of Rheumatology. For the *in vitro* experiments, PBMCs isolated from healthy donors were cultured in RPMI 1640 medium containing 10% FBS and stimulated with R837 (1 μg/ml, Enzo), CpG 2006S (0.5 μM, Invitrogen) or human IFN-α (1,000 U/ml, Biolegend) to detect CD180 expression. The study protocol was approved by the ethics committee at Jining Medical University.

### Preparation of bone marrow-derived macrophages (BMDMs)

BMDMs were obtained as previously described (38). Briefly, bone marrow cells, isolated from tibias and femurs of C57BL/6 mice, were cultured in complete DMEM medium supplemented with GM-CSF (10 ng/ml, Peprotech). After 4 days, the cells received a fresh complete DMEM medium supplemented with GM-CSF (10 ng/ml) and were incubated for 3 additional days. BMDMs were harvested and then seeded in fresh complete DMEM medium at a density of 2 × 10^6^ cells/ml for experiments. Under certain condition, anti-CD180 Ab (0.2 μg/ml) or IgG2b antibody (0.2 μg/ml) was added to BMDMs before stimulated with R837 (1 μg/ml, Enzo), CpG 1826 (0.5 μM, Invitrogen) or murine IFN-α (1,000 U/ml, Biolegend); to block the activity of SHP-1 and SHP-2 in BMDMs, the cells were pretreated with SHP-1 and SHP-2 inhibitor NSC-87877 (1 μM, SELLECK) for 30 min and then used for subsequent experiments. The vehicle used to delivery anti-CD180 antibody and IgG2b antibody is phosphate-buffered solution containing no preservative.

### Preparation of bone marrow-derived dendritic cells (BMDCs)

Bone marrow cells, isolated from tibias and femurs of C57BL/6 mice, were planted into dishes using complete RPMI 1640 medium supplemented with GM-CSF (20 ng/ml) and IL-4 (10 ng/ml, Peprotech) and maintained at 37°C in 5% CO_2_-humidified atmosphere. The cells received a fresh complete RPMI 1640 medium supplemented with GM-CSF (20 ng/ml) and IL-4 (10 ng/ml) on day 3 and day 5. On day 7, BMDCs were harvested and then seeded in fresh complete RPMI 1640 medium at a density of 2 × 106 cells/ml for experiments. Under certain condition, anti-CD180 antibody (0.2 μg/ml) or IgG2b antibody (0.2 μg/ml) was added to BMDCs before stimulated with R837 (1 μg/ml, Enzo), CpG 1826 (0.5 μM, Invitrogen), or murine IFN-α (1,000 U/ml, Biolegend); to block the activity of SHP-1 and SHP-2 in BMDCs, the cells were pretreated with NSC-87877 (1 μM, SELLECK) for 30 min and then used for subsequent experiments.

### Murine B-cell purification and cell culture

Mouse spleen lymphocytes were isolated by ficoll density centrifugation according to standard procedures. Mouse splenic B cells were purified using a mouse B cell isolation kit (Miltenyi Biotec) and the B-cell purity was always above 95%. Purified B cells were cultured in RPMI 1640 medium containing 10% FBS. Under certain condition, B cells were pretreated with anti-CD180 antibody (0.2 μg/ml) or IgG2b antibody (0.2 μg/ml) followed by stimulated with R837 (1 μg/ml) and CpG 1826 (0.5 μM).

### Flow cytometry

Cells were washed twice with phosphate buffered saline (PBS) containing 1% FBS and 0.1% NaN_3_. The cells were then surface-stained with mouse antibodies for 30 min at 4°C. After washed twice with PBS, the cells were analyzed by FACS Calibur (Becton Dickinson). An isotype control was used for each antibody. All the FACS data were analyzed on FlowJo software.

### RNA isolation and quantitative real-time RT–PCR (Q-PCR)

Total RNA was extracted using TRIzol reagent (Invitrogen) according to the manufacturer's instructions and then reverse-transcribed with a RevertAid First Strand cDNA Synthesis Kit (ThermoFisher Scientific). Q-PCR assays for mRNA were performed with a SYBR Green PCR Master Mix (Vazyme Biotech). The 2^−ΔΔ*Ct*^ method was used for real-time quantitative PCR gene expression analysis. All the expression levels of target genes were normalized to the β-actin level.

### Immunoblotting analysis

Cells were collected and homogenized in lysis buffer followed by centrifugation for 15 min at 12,000 g. The protein concentration was measured with a BCA protein assay kit (Beyotime). Proteins were separated by 10% SDS-PAGE and then transferred to 0.45 μm PVDF membranes (Millipore). Membranes were blocked in TBST containing 3% BSA for 2 h at room temperature and then incubated with primary antibodies overnight at 4°C. After washing, the membranes were incubated with HRP-conjugated secondary antibodies for 2 h at room temperature. ECL plus western blotting detection reagents (ThermoFisher Scientific) were used to visualize protein expression. β-actin was used as an internal control.

### Histological analyses

Histological analyses were performed as described previously (38). Briefly, sections were cut from paraffin-embedded kidney tissue, fixed in paraformaldehyde (Sigma), and stained with haematoxylin and eosin. The sections were scored by two professional renal pathologists for glomerular, interstitial, and vascular lesions according to reported criteria. In brief, the severity of renal lesions in murine lupus nephritis was graded on a semi-quantitative scale ranging from 0 to 4 as follows: 0 = normal; 1 = a small increase of cells in the glomerular mesanguim; 2 = a larger number of cells in the mesangium; 3 = glomerular lobular formation and thickened basement membrane; 4 = glomerular crescent formation, sclerosis, tubular atrophy, and casts. The score for each animal was calculated by dividing the total score for the number of glomeruli observed.

### Enzyme-linked immunosorbent assay

The secretions of IL-6, IL-12, TNF-α, and IL-1β in cell culture were determined by mouse ELISA kit (Biolegend) according to the standard procedure. Proteinuria was determined using a mouse albumin ELISA Quantitation Set (Bethyl Laboratories) according to the standard procedure. Anti-dsDNA IgG were analyzed by using mouse anti-dsDNA IgG Kit (Bethyl Laboratories) according to the standard procedure. Anti-RNA IgG ELISAs were performed as described (39). All samples were assayed in duplicate.

### Lentivirus infections

Lentiviruses expressing negative control-RNAi (NC-RNAi-LV) or Lyn-specific RNAi (Lyn-RNAi-LV) were purchased from GeneChem (China) and used to infect BMDMs and BMDCs following the standard protocols. Briefly, on day 3 during the induction of BMDMs or BMDCs, the cells were infected with 100 multiplicity of infection (MOI) NC-RNAi-LV or Lyn-RNAi-LV. After 24 h, stale medium were replaced by fresh medium with GM-CSF (10 ng/ml). On day 7, BMDMs and BMDCs were collected and used for subsequent experiments.

### Immunofluorescence staining

The direct immunofluorescence technique was performed on kidney sections using Alexa Fluor 488-conjugated goat anti-mouse IgG, Alexa Fluor 488-conjugated goat anti-mouse IgM (Invitrogen™). Briefly, a series of xylene and ethanol dewaxed and rehydrated tissue sections were used. After blocked with 1% BSA and exposed to Alexa Fluor 488-conjugated goat anti-mouse IgG or Alexa Fluor 488-conjugated goat anti-mouse IgM overnight at 4°C. Second day, the sections were washed in PBS with 0.1% Tween 20 for 5 times and then sealed the cover slips with anti-fluorescence quenching agent. The sections were analyzed under a fluorescence microscope (Olympus, Japan).

### Statistical analysis

All values in the graphs were given as means plus or minus standard error of the mean (SEM). To assess the statistical significance, ANOVA tests or unpaired Student's *t*-test were performed with GraphPad Prism 5.0. A statistical significance level was set at *p* < 0.05.

## Results

### CD180-negative monocytes and DCs are both increased in SLE patients

**Figure 1:**
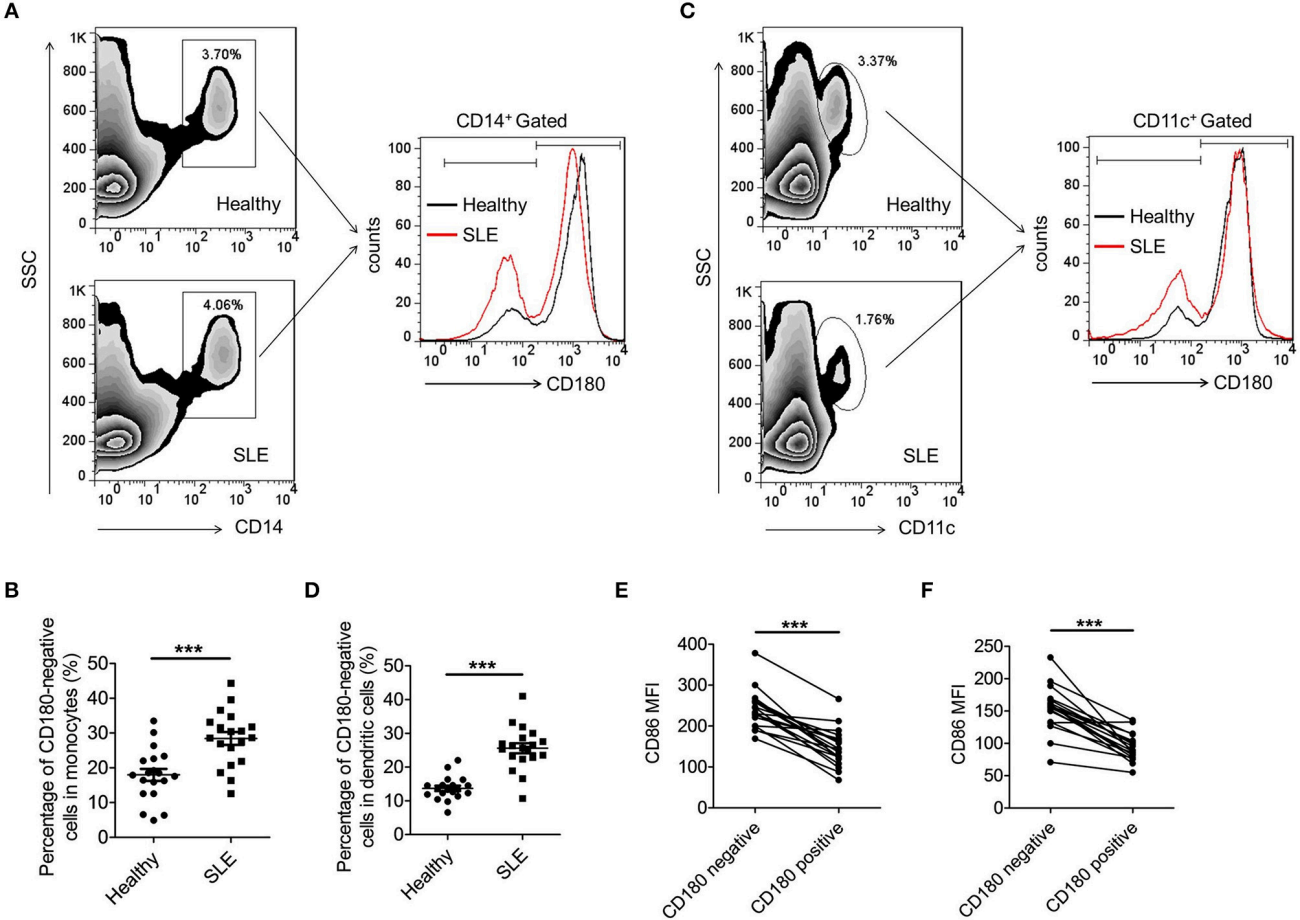
Increased percentages of CD180-negative monocytes and DCs in PBMCs from SLE patients. **(A,B)** Flow cytometric analysis of CD180 expression on CD14^+^ monocytes in PBMCs from SLE patients (*n* = 19) and healthy donors (*n* = 19). **(C,D)** Flow cytometric analysis of CD180 expression on CD11c^+^ DCs in PBMCs from SLE patients (*n* = 19) and healthy donors (*n* = 19). **(E)** Flow cytometric analysis of CD86 expression on CD180-negative and CD180-positive monocytes population in PBMCs from SLE patients (*n* = 19) and healthy donors (*n* = 19). **(F)** Flow cytometric analysis of CD86 expression on CD180-negative and CD180-positive DCs population in PBMCs from SLE patients (*n* = 19) and healthy donors (*n* = 19). Error bars represent S.E.M. ***p < 0.001, as determined by *t*-test.

Our previous study and others have reported that the percentage of CD180-negative B cells is dramatically increased in PBMCs from SLE patients (32, 33). However, the mode of CD180 expression on monocytes and DCs in SLE patients is still unknown. As shown in Figures 1A,B, the percentage of CD180-negative monocytes in PBMCs from SLE patients was significantly increased compared with that from healthy donors. More than this, SLE patients also showed increased percentage of CD180-negative DCs in PBMCs compared with healthy donors (Figures 1C,D). Since abnormal expression of CD180 on monocytes and DCs exists in SLE patients, we further investigated the molecular events that lead to CD180 downregulation. It is known that TLR7, TLR9, and IFN-α pathways play vital roles in the pathogenesis of SLE, we therefore evaluated the effects of these pathways on CD180 expression in human PBMCs. Interestingly, the challenges of TLR7 ligand R837 and TLR9 ligand CpG 2006S, but not IFN-α, could significantly decrease CD180 expression in human PBMCs (Figure S1), indicating that increased percentages of CD180-negative monocytes and DCs in SLE patients maybe a result of hyper-activation of TLR7 and TLR9 pathways.

As is known, immune cells, including monocytes and DCs, were excessively activated in SLE. To investigate whether CD180 was related with the activation of monocytes and DCs, we detected the activation markers CD86 on CD180-negative and CD180-positive monocytes, as well as CD180-negative and CD180-positive DCs. Intriguingly, CD180-negative monocytes showed a significantly higher level of CD86 than that on CD180-positive monocytes (Figure 1E). Moreover, a significant increase of CD86 expression was also detected on CD180-negative DCs compared with that on CD180-positive DCs (Figure 1F). All these data demonstrate that the percentages of CD180-negative monocytes and DCs are both increased in SLE patients and the expression of CD180 was negatively correlated with the activation of monocytes and DCs, indicating that CD180 may play a negatively role in regulating the activation of monocytes and DCs.

### CD180-negative macrophages and DCs are both increased in lupus-prone MRL/*lpr* mice

**Figure 2:**
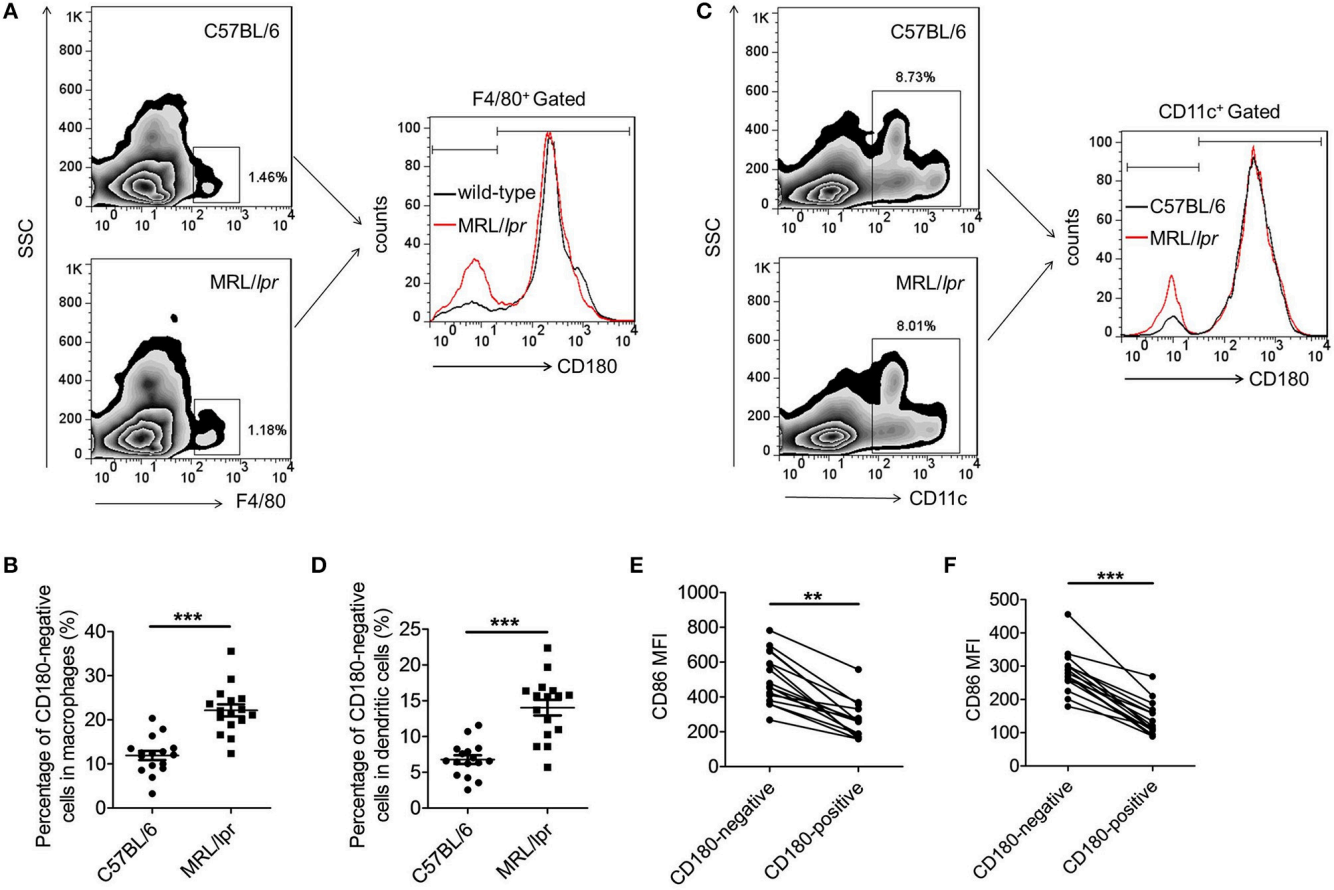
Increased percentages CD180-negative macrophages and DCs in spleens from MRL/*lpr* mice. **(A,B)** Flow cytometric analysis of CD86 expression on F4/80^+^ macrophages in spleens from 18-week MRL/*lpr* mice (*n* = 16) and control C57BL/6 mice (*n* = 16). **(C,D)** Flow cytometric analysis of CD86 expression on CD11c^+^ DCs in spleens from 18-week MRL/*lpr* mice (*n* = 16) and control C57BL/6 mice (*n* = 16). **(E)** Flow cytometric analysis of CD86 expression on CD180-negative and CD180-positive macrophages population in spleens from 18-week MRL/*lpr* mice (*n* = 16) and control C57BL/6 mice (*n* = 16). **(F)** Flow cytometric analysis of CD86 expression on CD180-negative and CD180-positive DCs in spleens from 18-week MRL/*lpr* mice (*n* = 16) and control C57BL/6 mice (*n* = 16). Error bars represent S.E.M. **p < 0.01, ***p < 0.001, as determined by *t*-test.

To investigate whether the above phenomenon in SLE patients could also extend to lupus-prone mice, we then evaluated the percentages of CD180-negative macrophages and DCs in the spleens from MRL/*lpr* mice and control C57BL/6 mice. As shown in Figures 2A,B, the percentage of CD180-negative macrophages in the spleens from MRL/*lpr* mice was significantly increased compared with that from control C57BL/6 mice. Moreover, MRL/*lpr* mice also showed an increased percentage of CD180-negative DCs in spleens compared with control C57BL/6 mice (Figures 2C,D). In addition, we investigated the effects of TLR7, TLR9, and IFN-α pathways in regulating CD180 expression on murine macrophages and DCs. Consistent with the result in Figure S1, TLR7 ligand R837 and TLR9 ligand CpG 1826, but not IFN-α, could downregulate the expression of CD180 in murine BMDMs (Figures S2A,B) and BMDCs (Figures S2C,D).

Evaluation of activation markers CD86 on CD180-negative and CD180-positive macrophages, as well as CD180-negative and CD180-positive DCs, revealed that the expressions of CD86 on CD180-negative macrophages (Figures 2E) and CD180-negative DCs (Figure 2F) were significantly higher than that on CD180-positive macrophages and CD180-positive DCs, respectively. Collectively, all these data indicate that CD180-negative macrophages and DCs are both increased in lupus-prone MRL/*lpr* mice and CD180 expression were negatively correlated with the activation of macrophages and DCs.

### Ligation of CD180 by anti-CD180 Ab inhibits TLR7- and TLR9-mediated activation of macrophages

**Figure 3:**
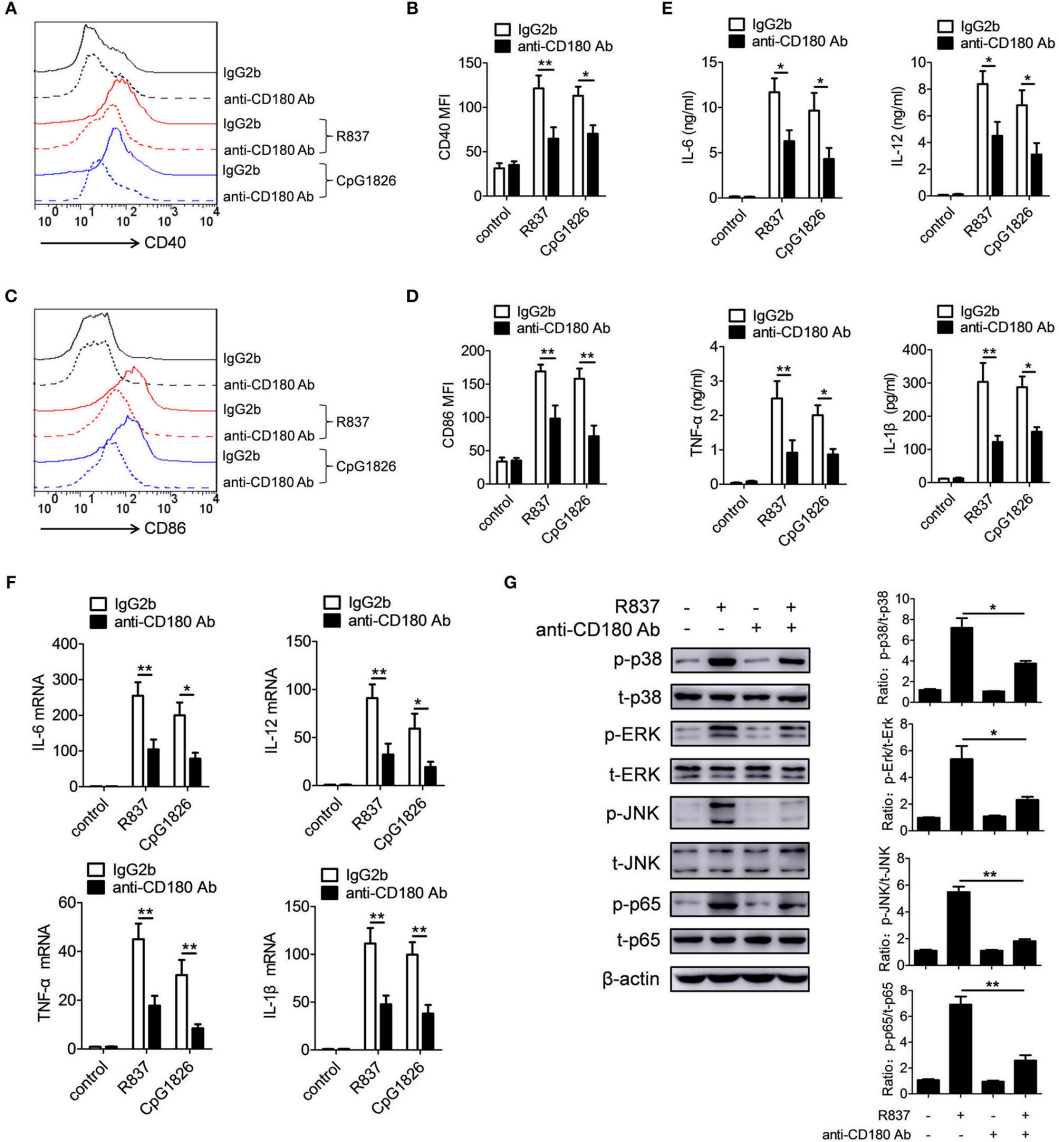
Ligation of CD180 inhibits TLR7- and TLR9-mediated activation of macrophages. BMDMs were pretreated with anti-CD180 antibody (0.2 μg/ml) or IgG2b antibody (0.2 μg/ml) followed by stimulation of R837 (1 μg/ml) and CpG1826 (0.5 μM). **(A,B)** Flow cytometric analysis of CD40 expression at 24 h. **(C,D)** Flow cytometric analysis of CD86 expression at 24 h. **(E)** ELISA analysis of secretions of IL-6, IL-12, TNF-α and IL-1β in cell culture at 24 h. **(F)** Q-PCR analysis of the mRNA levels of IL-6, IL-12, TNF-α, and IL-1β at 6 h. The axis labels means the fold differences over control. **(G)** Western blot analysis of the phosphorylation levels of p38, Erk, JNK, and p65 at 1 h. The data shown represent the means of three independent experiments and the error bars represent the S.E.M. *p < 0.05, **p < 0.01, as determined by ANOVA tests. The control to R837 and CpG 1826 was untreated control.

Previous studies have shown that CD180 negatively regulates TLR4-mediated activation of macrophages (34). However, little is known about the effect of CD180 on TLR7- and TLR9-mediated activation of macrophages. To identify a potential role for CD180 in the activation of TLR7 and TLR9 pathways, BMDMs were pretreated with anti-CD180 Ab for 1 h followed by stimulations of R837 and CpG 1826, and then the expression of CD86 and pro-inflammatory cytokines were measured. As shown in Figures 3A,B, addition of exogenous anti-CD180 Ab alone showed no effect on the basal expression of CD40, while anti-CD180 Ab could significantly inhibit R837 and CpG 1826-induced expression of CD40 on BMDMs. Similar phenomenons were also found in the expression of CD86 on BMDMs (Figures 3C,D). Not only that, anti-CD180 Ab could significantly reduce the secretions of IL-6, IL-12, TNF-α and IL-β by BMDMs induced by R837 and CpG 1826 (Figure 3E). Q-PCR analysis also showed that anti-CD180 Ab treatment could inhibit R837 and CpG 1826-induced mRNA levels of IL-6, IL-12, TNF-α, and IL-β in BMDMs (Figure 3F). To better understand the role of CD180 on activation of TLR7 pathway, we next examined the effect of anti-CD180 Ab on R837-activated MAPKs and NF-κB pathways. In agreement with the above results, anti-CD180 Ab could significantly inhibit R837-induced phosphorylation of p38, Erk, JNK, and p65 (Figure 3G). Collectively, these data demonstrate that ligation of CD180 can indeed inhibit TLR7- and TLR9-mediated activation of macrophages.

### Ligation of CD180 by Anti-CD180 Ab inhibits TLR7- and TLR9-mediated activation of DCs

**Figure 4:**
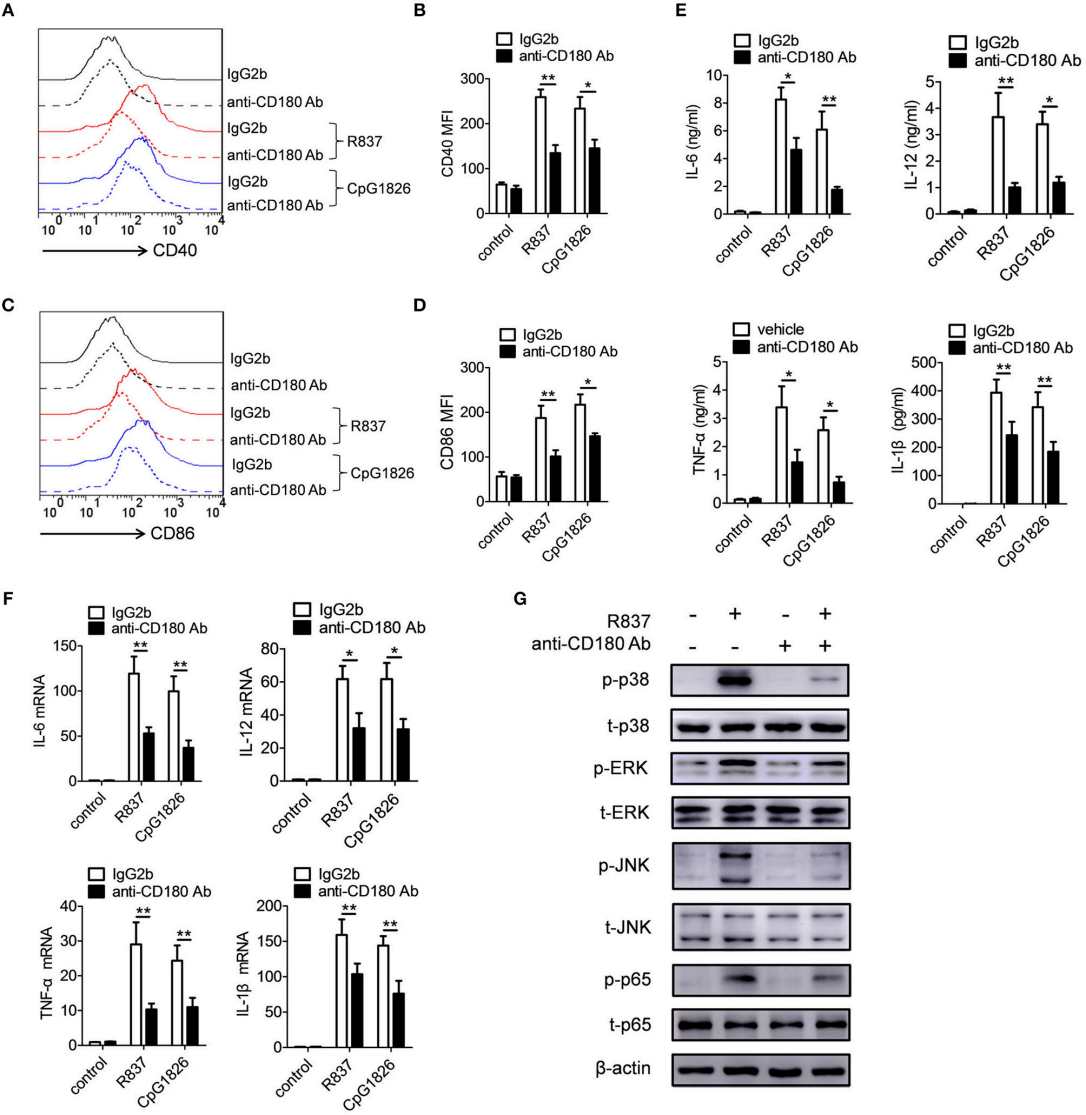
Ligation of CD180 inhibits TLR7- and TLR9-mediated activation of DCs. BMDCs were pretreated with anti-CD180 antibody (0.2 μg/ml) followed by stimulation of R837 (1 μg/ml) and CpG 1826 (0.5 μM). **(A,B)** Flow cytometric analysis of CD40 expression at 24 h. **(C,D)** Flow cytometric analysis of CD86 expression at 24 h. **(E)** ELISA analysis of secretions of IL-6, IL-12, TNF-α, and IL-1β in cell culture at 24 h. **(F)** Q-PCR analysis of the mRNA levels of IL-6, IL-12, TNF-α, and IL-1β at 6 h. The axis label means the fold differences over control. **(G)** Western blot analysis of the phosphorylation levels of p38, Erk, JNK, and p65 at 1 h. The data shown represent the means of three independent experiments and the error bars represent the S.E.M. *p < 0.05, **p < 0.01, as determined by ANOVA tests. The control to R837 and CpG 1826 was untreated control.

We also evaluated the effect of CD180 on the activation of TLR7 and TLR9 pathways in BMDCs. BMDCs were pretreated with anti-CD180 Ab for 1 h followed by stimulations of R837 and CpG 1826, and then the expression of CD86 and proinflammatory cytokines were analyzed. Intriguingly, we obtained a similar effect of CD180 on the activation of TLR7 and TLR9 pathways in BMDCs. As shown in Figures 4A,B, addition of exogenous anti-CD180 Ab resulted in an decrease in R837 and CpG 1826-induced expression of CD40 on BMDCs, as well as the expression of CD86 (Figures 4C,D). What's more, anti-CD180 Ab could reduce R837 and CpG 1826-induced secretions of IL-6, IL-12, TNF-α, and IL-β by BMDCs (Figure 4E). Q-PCR analysis also showed that anti-CD180 Ab could inhibit R837 and CpG 1826-induced mRNA levels of IL-6, IL-12, TNF-α, and IL-β in BMDCs (Figure 4F). We then sought to determine whether anti-CD180 Ab could affect the activation of MAPKs and NF-κB pathways induced by R837 in BMDCs and found that anti-CD180 Ab could significantly inhibit R837-induced phosphorylation of p38, Erk, JNK, and p65 (Figure 4G). Taken together, these data demonstrate that ligation of CD180 can indeed inhibit TLR7- and TLR9-mediated activation of DCs.

### The lyn-SHP-1/2 axis is required for the inhibitory effect of CD180 on the activation of TLR7 and TLR9 pathways

**Figure 5:**
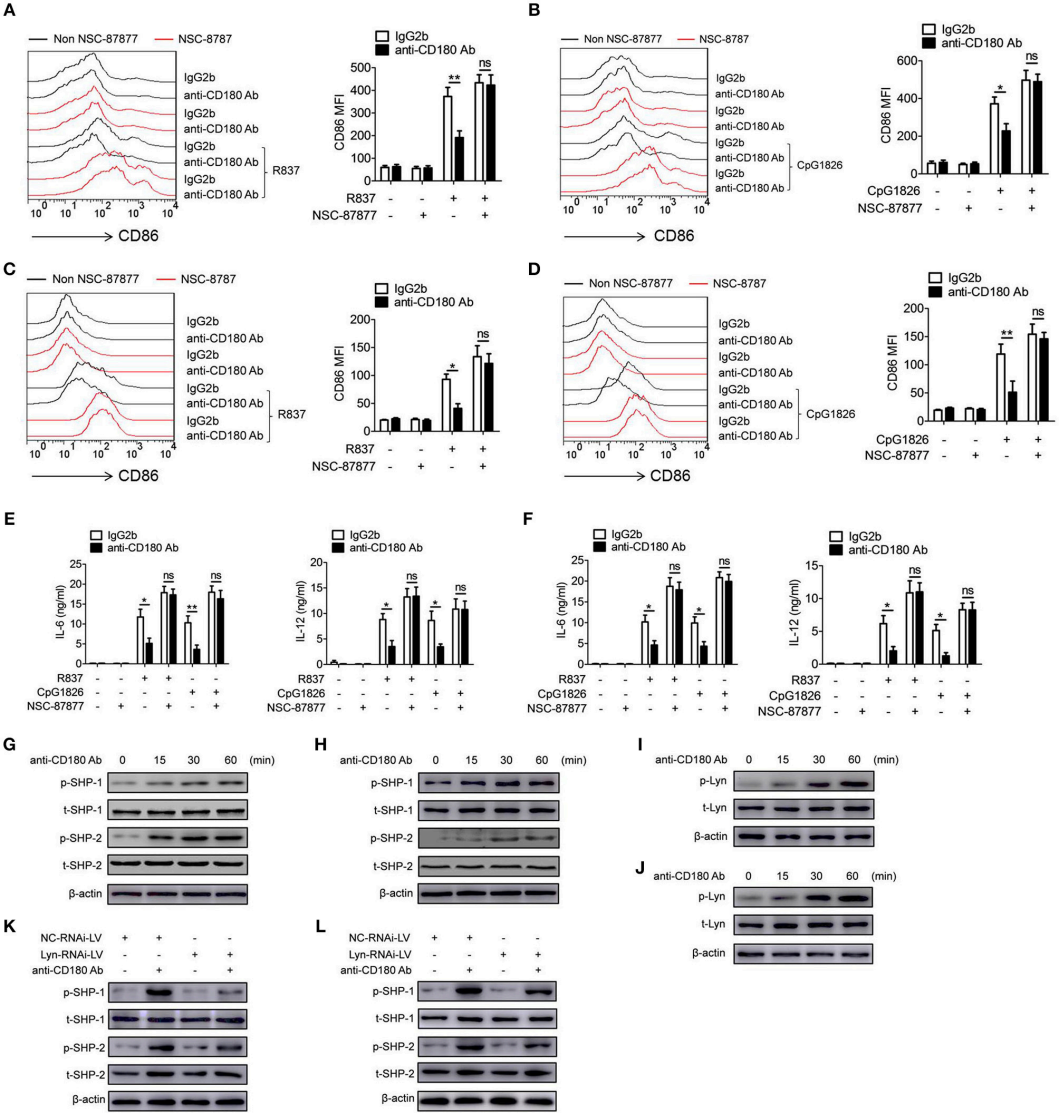
Lyn-SHP-1/2 axis is required for the inhibitory effect of CD180 on TLR7- and TLR9-mediated activation of macrophages and DCs. **(A–F)** BMDMs and BMDCs were pretreated with NSC87877 (1 μM) for 1 h followed by stimulation with anti-CD180 Ab (0.2 μg/ml). Then the cells were stimulated with R837 (1 μg/ml) and CpG 1826 (0.5 μM). Flow cytometric analysis of CD86 expression on BMDMs **(A,B)** and BMDCs **(C,D)** at 24 h. ELISA analysis of the levels of IL-6 and IL-12 secreted by BMDMs **(E)** and BMDCs **(F)** at 24 h. **(G,H)** BMDMs **(G)**, or BMDCs **(H)** were stimulated with anti-CD180 antibody (0.2 μg/ml) for different times and western blot analysis of the phosphorylation levels of SHP-1 and SHP-2. **(I,J)** BMDMs **(I)** or BMDCs **(J)** were stimulated with anti-CD180 antibody (0.2 μg/ml) for different times and western blot analysis of the phosphorylation levels of Lyn. **(K,L)** BMDMs **(K)** and BMDCs **(L)**, which have been infected with lentivirus expressing negative control-RNAi (NC-RNAi-LV) or Lyn-specific RNAi (Lyn-RNAi-LV), were stimulated with anti-CD180 antibody (0.2 μg/ml) for different times and western blot analysis of the phosphorylation levels of SHP-1 and SHP-2. The data shown represent the means of three independent experiments and the error bars represent the S.E.M. *p < 0.05, **p < 0.01, as determined by ANOVA tests; ns denotes *p* > 0.05.

Given that SHP-1 and SHP-2 play negative roles in regulating TLRs pathways (23–25), we next assessed whether SHP-1 and SHP-2 were involved in the inhibitory function of CD180 on TLR7- and TLR9-mediated activation of macrophages and DCs. We first confirmed the roles of SHP-1 and SHP-2 in TLR7- and TLR9-mediated activation of macrophages and DCs. As shown in Figure S3, inhibition of the activities of SHP-1 and SHP-2 by NSC-87877 could significantly promote R837- and CpG 1826-induced secretions of IL-6 and IL-12 by BMDMs (Figures S3A,B) and BMDCs (Figures S3C,D), indicating the involvement of SHP-1 and SHP-2 in TLR7- and TLR9-mediated activation of macrophages and DCs. To determine whether SHP-1 and SHP-2 participate in CD180-mediated inhibition of TLR7 and TLR9 pathways, BMDMs and BMDCs were pretreated with NSC-87877 or vehicle for 30 min before treated with anti-CD180 Ab. Two hours later, the cells were stimulated with R837 and CpG 1826. Intriguingly, NSC-87877 treatment could markedly reverse the effect of CD180 on inhibiting R837 and CpG 1826-induced CD86 expression on BMDMs (Figures 5A,B) and the secretions of IL-6 and IL-12 by BMDMs (Figure 5E). The same phenomenon was also found in the expression of CD86 on BMDCs (Figures 5C,D) and the secretions of IL-6 and IL-12 by BMDCs (Figure 5F). Importantly, we investigated the effect of anti-CD180 Ab on the phosphorylation of SHP-1 and SHP-2 and immunoblotting analysis showed that ligation of CD180 by anti-CD180 Ab could significantly induce the phosphorylation of SHP-1 and SHP-2 in BMDMs (Figure 5G) and BMDCs (Figure 5H).

Our previous study and others have reported that ligation of CD180 can activate Lyn and subsequently induce the activation of downstream signaling pathways (28, 32). To gain mechanistic insight into how CD180 ligation leads to the phosphorylation of SHP-1 and SHP-2, we next investigated whether CD180 induced the phosphorylation of SHP-1 and SHP-2 through Lyn. As shown in Figures 5I,J, ligation of CD180 by anti-CD180 Ab could significantly induce the phosphorylation of Lyn in BMDMs and BMDCs. To better identify the role of Lyn in CD180-induced phosphorylation of SHP-1 and SHP-2, knockdown of Lyn was performed in BMDMs and BMDCs. As shown in Figure S4, BMDMs and BMDCs infected with Lyn-RNAi-LV showed significantly lower level of Lyn compared with that infected with NC-RNAi-LV. Importantly, immunoblotting analysis showed that knockdown of Lyn could significantly inhibit CD180-induced phosphorylation of SHP-1 and SHP-2 in BMDMs (Figure 5K) and BMDCs (Figure 5L). From these experiments, we concluded that CD180, at least in large part, inhibited R848 and CpG 1826-induced activation of macrophages and DCs through the Lyn-SHP-1/2 axis.

### Ligation of CD180 ameliorates lupus-like symptoms in IMQ-mice

**Figure 6:**
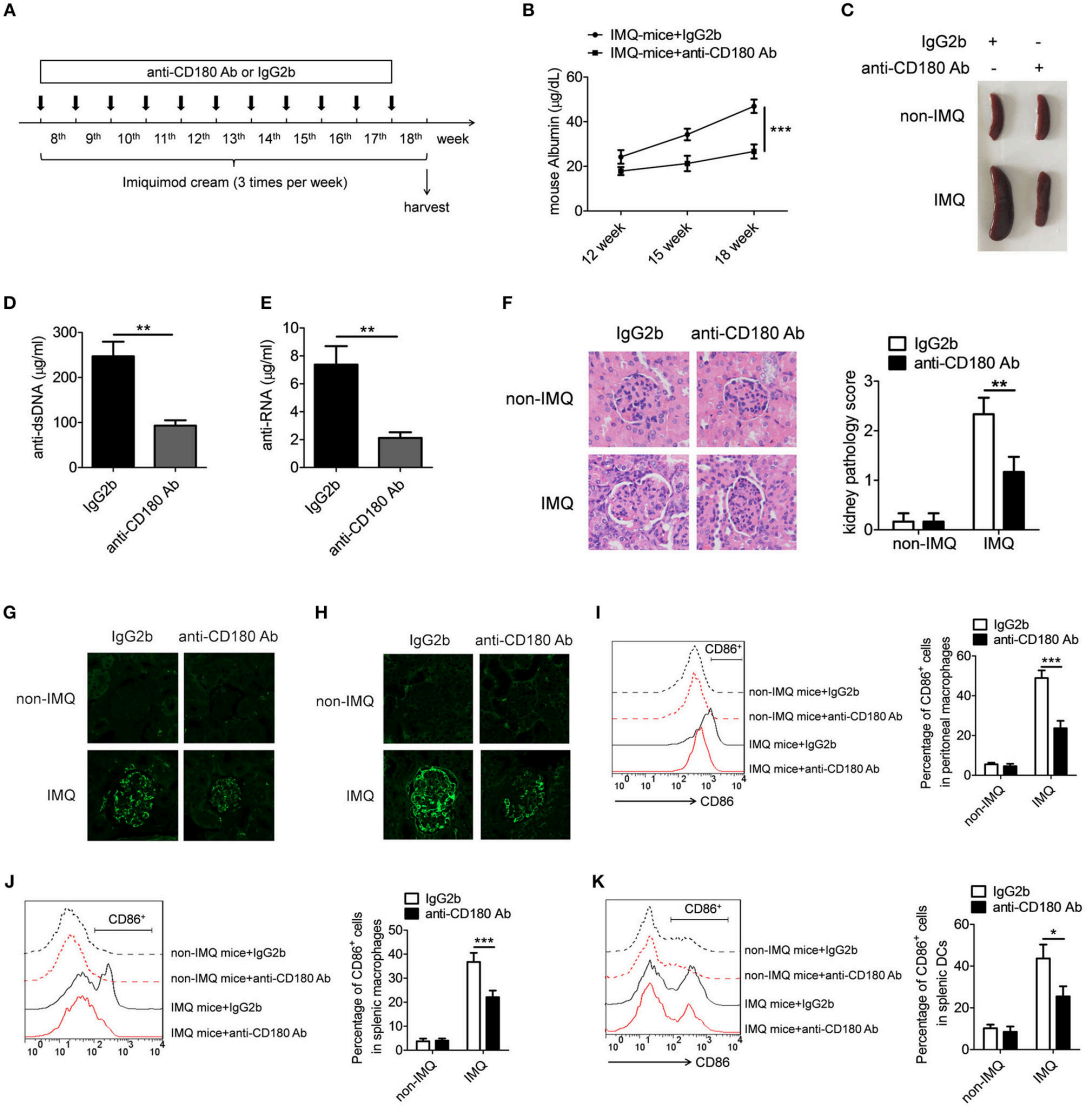
Treatment effect of anti-CD180 Ab on lupus-symptoms of IMQ-treated mice. **(A)** Treatment schematic of anti-CD180 Ab and IgG2b injection to mice following epicutaneous application of the TLR7 agonist imiquimod. **(B)** Proteinuria was collected weekly and determined by ELISA. **(C)** Representative images of marked splenomegaly in all groups of mice. **(D,E)** ELISA analysis of the anti-dsDNA antibody **(D)** and anti-RNA antibody **(E)** in serum. **(F)** H&E staining of the kidney sections from all groups of mice. **(G)** Immunofluorescence staining to detect IgG deposit in the kidney sections. **(H)** Immunofluorescence staining to detect IgM deposit in the kidney sections. **(I–K)** Flow cytometric analysis of CD86 expression on peritoneal macrophages **(I)**, splenic macrophages **(J)**, and DCs **(K)** in all groups of mice. The data are shown as the means ± SEM (*n* = 7 mice/group). *p < 0.05, **p < 0.01, ***p < 0.001, as determined by ANOVA tests or *t*-test.

Since our earlier data showed that ligation of CD180 can negatively regulate TLR7- and TLR9-mediated activation of macrophages and DCs *in vitro*, we guess that ligation of CD180 may inhibit the activation of macrophages and DCs *in vivo*, and relieve the symptoms of lupus-prone mice. To test this hypothesis, we investigated the treatment effect of anti-CD180 Ab and IgG2b on lupus-symptoms in mice following epicutaneous application of the TLR7 agonist imiquimod (IMQ-mice) according to the treatment schematic shown in Figure 6A. We monitored the levels of proteinuria and found that severe increase of proteinuria was existed in IgG2b-treated IMQ-mice compared with control mice, while the proteinuria level in anti-CD180 Ab-treated IMQ-mice was significantly lower than that in IgG2b-treated IMQ-mice (Figure 6B). Imiquimod could induce marked splenomegaly in IMQ-mice, while imiquimod-induced splenomegaly was significantly reversed in anti-CD180 Ab-treated IMQ-mice (Figure 6C). Assessment of serum levels of anti-dsDNA antibody and anti-RNA antibody revealed that anti-CD180 Ab-treated IMQ-mice showed decreased amount of anti-dsDNA antibody and anti-RNA antibody compared with IgG2b-treated IMQ-mice (Figures 6D,E). H&E staining showed that there was significant inhibition in the infiltration of lymphoid cells as well as the diffuse expansion of the mesangial matrix in the kidneys of anti-CD180 Ab-treated IMQ-mice when compared with that in IgG2b-treated IMQ-mice (Figure 6F). Immunofluorescence staining of the kidney tissue revealed that, compared with IgG2b-treated IMQ-mice, there were less glomerular deposition of IgG (Figure 6G) and IgM (Figure 6H) in anti-CD180 Ab-treated IMQ-mice.

We were also interested in whether anti-CD180 Ab influences the activation of macrophages and DCs in IMQ-mice. Strikingly, elevated levels of activation marker CD86 were observed in peritoneal macrophages, splenic macrophages and DCs from IgG2b-treated IMQ-mice compared with control mice, while anti-CD180 Ab treatment could markedly reverse IMQ-induced CD86 expression on peritoneal macrophages (Figure 6I), splenic macrophages (Figure 6J), and DCs (Figure 6K), indicating that ligation of CD180 can inhibit activation of macrophages and DCs *in vivo*. What's more, the effect of anti-CD180 Ab on the activation of B cells was detected. Interestingly, anti-CD180 Ab treatment could also significantly inhibit the activation of splenic B cells from IMQ-mice (Figure S5). However, ligation of CD180 by anti-CD180 Ab showed no effect on TLR7- and TLR9-mediated activation of B cells *in vitro* (Figure S6), hinting that the inhibitory function of anti-CD180 Ab on the activation of B cells *in vivo* may be related with reduced activation of macrophages and DCs. Collectively, all these data suggest that ligation of CD180 by anti-CD180 Ab show an efficient treatment effect on lupus-like symptoms in IMQ-mice.

### Ligation of CD180 ameliorates lupus-symptoms in lupus-prone MRL/*lpr* mice

**Figure 7:**
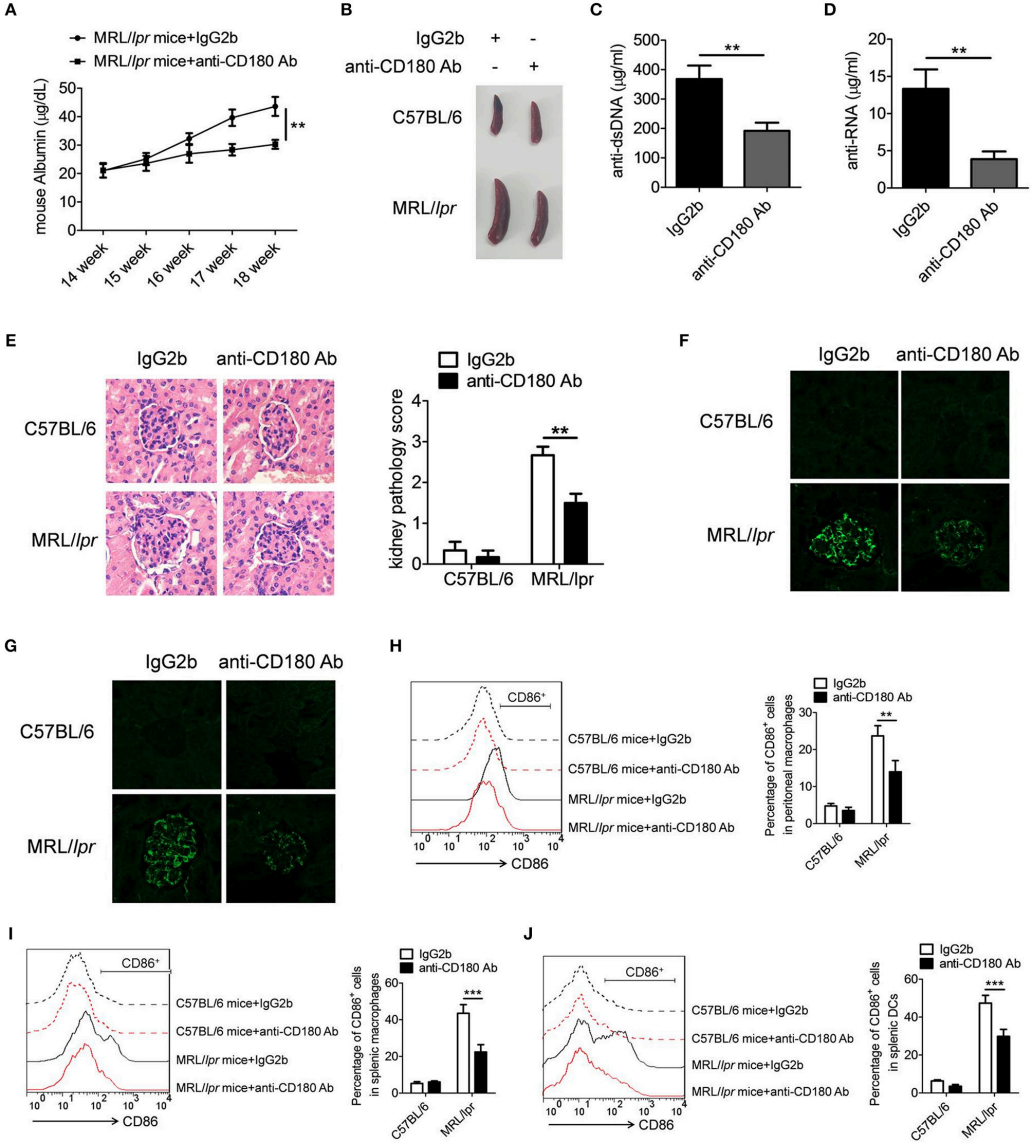
Treatment effect of anti-CD180 Ab on lupus-symptoms of MRL/*lpr* mice. **(A)** Proteinuria was collected weekly and determined by ELISA. **(B)** Representative images of marked splenomegaly in all groups of mice. **(C,D)** ELISA analysis of the anti-dsDNA antibody **(C)** and anti-RNA antibody **(D)** in serum. **(E)** H&E staining of the kidney sections from all groups of mice. **(F)** Immunofluorescence staining to detect IgG deposit in the kidney sections. **(G)** Immunofluorescence staining to detect IgM deposit in the kidney sections. **(H–J)** Flow cytometric analysis of CD86 expression on peritoneal macrophages **(H)**, splenic macrophages **(I)**, and DCs **(J)** in all groups of mice. The data are shown as the means ± SEM (*n* = 6 mice/group). **p < 0.01, ***p < 0.001, as determined by ANOVA tests or *t*-test.

To further confirm the above phenomenon, we investigated the treatment effect of anti-CD180 Ab and IgG2b on the lupus-symptoms in MRL/*lpr* mice and consistent results were obtained. As shown in Figure 7A, severe increase of proteinuria was observed in the IgG2b-treated MRL/*lpr* mice compared with control C57BL/6 mice, while the proteinuria level in anti-CD180 Ab-treated MRL/*lpr* mice was significantly lower than that in IgG2b-treated MRL/*lpr* mice. Compared with control C57BL/6 mice, IgG2b-treated MRL/*lpr* mice displayed marked splenomegaly, while splenomegaly was significantly reversed in anti-CD180 Ab-treated MRL/*lpr* mice (Figure 7B). Moreover, compared with the IgG2b-treated MRL/*lpr* mice, anti-CD180 Ab-treated MRL/*lpr* mice showed lower levels of anti-dsDNA antibody and anti-RNA antibody in serum (Figures 7C,D). H&E staining showed that there was significant inhibition in the infiltration of lymphoid cells as well as the diffuse expansion of the mesangial matrix in the kidneys of anti-CD180 Ab-treated MRL/*lpr* mice when compared with that in IgG2b-treated MRL/*lpr* mice (Figure 7E). Immunofluorescence staining of the kidney tissue revealed that, compared with IgG2b-treated MRL/*lpr* mice, there were less glomerular deposition of IgG (Figure 7F) and IgM (Figure 7G) in anti-CD180 Ab-treated MRL/*lpr* mice.

We then investigated whether anti-CD180 Ab influences the activation of macrophages and DCs in MRL/*lpr* mice. As expected, injection of anti-CD180 Ab could significantly reduce the levels of CD86 on peritoneal macrophages (Figure 7H), splenic macrophages (Figure 7I) and DCs (Figure 7J) from MRL/*lpr* mice, indicating that ligation of CD180 can inhibit the activation of macrophages and DCs *in vivo* and ameliorates lupus-symptoms in MRL/*lpr* mice. What's more, anti-CD180 Ab treatment could significantly inhibit the activation of splenic B cells from MRL/*lpr* mice (Figure S7), which is consistent with the result in Figure S5. Overall, these findings reveal an important role of CD180 in the pathogenesis of SLE and CD180 can be considered as a target in the treatment of SLE.

## Discussion

In the present study, we investigated the role of CD180 in regulating the activation of macrophages and DCs induced by ligands of TLR7 and TLR9. The data presented in this paper demonstrated that CD180, low-expressed in macrophages and DCs from SLE patients and lupus-prone MRL/*lpr* mice, could negatively regulate TLR7- and TLR9-mediated activation of macrophages and DCs *in vivo* and *in vitro*. What's more, we also noted that ligation of CD180 could significantly relieve the lupus-symptoms of IMQ-treated mice and MRL/*lpr* mice. Thus, for all intents and purposes, these findings provided validation for our working hypothesis.

As the percentages of CD180-negative macrophages and DCs were both increased in SLE patients and lupus-prone MRL/*lpr* mice, it is curious to explore the influencing factors which can induce the differentiation of these populations. Interestingly, we found that ligands of TLR7 and TLR9 significantly downregulated CD180 expression in human PBMCs, as well as murine macrophages and DCs. Combine with our previous study that the ligands of TLR7 and TLR9 could downregulate CD180 expression in B cells (32), we conclude that TLR7 and TLR9 may play critical roles in inducing the differentiation of CD180-negative macrophages and DCs. However, the specific mechanism underlying TLR7- and TLR9-mediated inhibition of CD180 expression remains largely unclear. Given that CD180 negatively regulates the activation of TLR7 and TLR9 pathways, downregulation of CD180 by TLR7 and TLR9 pathways can be considered as a candidate feedback mechanism which can regulate the activations of TLR pathways.

As is well-known, TLRs play critical roles in regulating the activation of immune cells and subsequently contribute to the development of SLE. However, the effect of CD180 on TLRs-mediated activation of immune cells was still largely unclear. We found that CD180 played a negative role in regulating TLR7- and TLR9-mediated activations of macrophages and DCs through Lyn-SHP-1/2 axis. In fact, Lyn has been reported to participate in the development of autoimmune diseases: Lyn can suppress TLR-MyD88 pathway to restrain the development of autoimmunity (40); B cells from SLE patients show decreased Lyn expression which can influence the B cell receptor signaling and B cell hyperactivity (41, 42). Consistently, we found that Lyn played a negative role in regulating the activations of TLR7 and TLR9 pathways. Notably, the expression of Lyn was interfered by using lentivirus system to explore the effect of Lyn in our study. However, the effect of interference was not very efficiency. Indeed, we think that it may be more conducive to study the role of Lyn in CD180-mediated activation of TLR7 and TLR9 pathways by constructing a Lyn-knockout mouse model.

We found that injection of anti-CD180 Ab could markedly relieve the lupus-symptoms of IMQ-treated mice and MRL/*lpr* mice. As is well-known, B cells play a critical role in the development of SLE. Thus, the effect of CD180 on the activation of B cells was also explored *in vivo* and *in vitro*. Interestingly, ligation of CD180 significantly inhibited the activation of B cells *in vivo*, which is consistent with the reduced levels of anti-dsDNA and anti-RNA antibody in CD180-treated IMQ-mice and MRL/*lpr* mice. However, *in vitro* study showed that ligation of CD180 could not directly inhibit TLR7- and TLR9-mediated activation of B cells, hinting that ligation of CD180 may indirectly inhibit the activation of B cells *in vivo* through other mechanisms. Since some studies have reported that ligation of CD180 can promote BCR-mediated apoptosis of B cells (43), we thought that the seemingly contradicting data on B cells *in vivo* and *in vitro* may be due to the BCR-TLR crosstalk *in vivo*. What's more, we found that ligation of CD180 showed no effect on the percentages of splenic B cells in IMQ-mice and MRL/*lpr* mice (Data not shown). The reason maybe that immune system is very complex and the survival of B cells *in vivo* can be regulated by many different signaling pathways. What's more, in order to really make the point that anti-CD180 Ab treatment relieves the lupus-symptoms of IMQ-treated mice and MRL/*lpr* mice through regulating the activations of macrophages and DCs, it needs to use a cell type specific knockout system, such as the MRL/*lpr* mice lacking myeloid cells. Meanwhile, we thought that further studies are needed to identify the effect of anti-CD180 Ab on the clinical treatment of SLE patients.

In conclusion, our studies demonstrate that the percentages of CD180-negative macrophages and DCs in SLE patients and lupus-prone MRL/*lpr* mice were significantly increased. Ligation of CD180 inhibits TLR7- and TLR9-mediated activation of macrophages and DCs through the Lyn-SHP-1/2 axis, and subsequently relieves the lupus-symptoms of lupus-prone mice. Taken together, our findings suggest a novel role of CD180 in regulating TLR7- and TLR9-mediated immune activation and CD180 may be used as a potential target in the treatment of SLE.

## Ethics statement

This study was carried out in accordance with the recommendations of Guide for the Care and Use of Laboratory Animals of Jining. Medical University and Animal Care Committee at Jining Medical University. The protocol was approved by the Animal Care Committee at Jining Medical University. All procedures were performed under sodium pentobarbital anesthesia, and all efforts were made to minimize suffering of the animals.

## Author contributions

HX, GD, and FH designed and supervised the study. YY, CW, PC, XZ, XL, YH, FX, and GD performed the experiments. YY and YH analyzed the data and wrote the paper.

### Conflict of interest statement

The authors declare that the research was conducted in the absence of any commercial or financial relationships that could be construed as a potential conflict of interest.
