# Supplementary material for: CD180 Ligation Inhibits TLR7- and TLR9-Mediated Activation of Macrophages and Dendritic Cells Through the Lyn-SHP-1/2 Axis in Murine Lupus
Source: Front Immunol. 2018 Nov 15;9:2643. doi: 10.3389/fimmu.2018.02643 (PMC6249336; doi:10.3389/fimmu.2018.02643)
Supplement: Supplementary file 1 [file Data_Sheet_1.PDF]

Supplemental Data

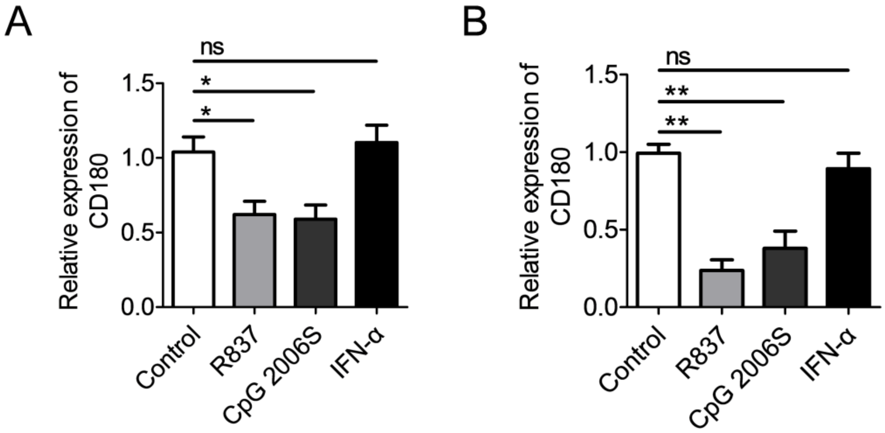

Figure S1 The effect of TLR ligands and human IFN-α on expression of CD180 in human PBMCs

Human PBMCs were stimulated with R837 (1 μg/ml), CpG 2006S (0.5 μM) and human IFN-α. Q-PCR analysis of the expression of CD180 at 6 hours **(A)** and 12 hours **(B)**. The data shown represent the means of three independent experiments and the error bars represent the S.E.M. * $p < 0.05$, ** $p < 0.01$, as determined by ANOVA tests; ns denotes $p > 0.05$.

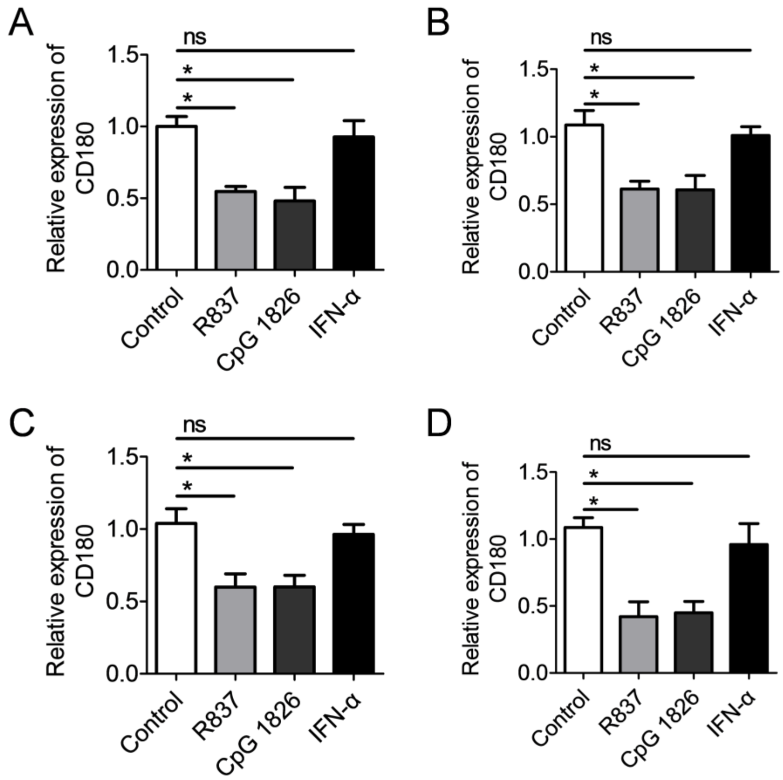

Figure S2 The effect of TLR ligands and murine IFN-α on expression of CD180 in BMDMs and BMDCs

Murine BMDMs and BMDCs were stimulated with R837 (1 μg/ml), CpG 1826 (0.5 μM) and murine IFN-α. **(A,B)** Q-PCR analysis of the expression of CD180 on BMDMs at 6 hours **(A)** and 12 hours **(B)**. **(C,D)** Q-PCR analysis of the expression of CD180 on BMDCs at 6 hours **(C)** and 12 hours **(D)**. The data shown represent the means of three independent experiments and the error bars represent the S.E.M. * $p < 0.05$, as determined by ANOVA tests; ns denotes $p > 0.05$.

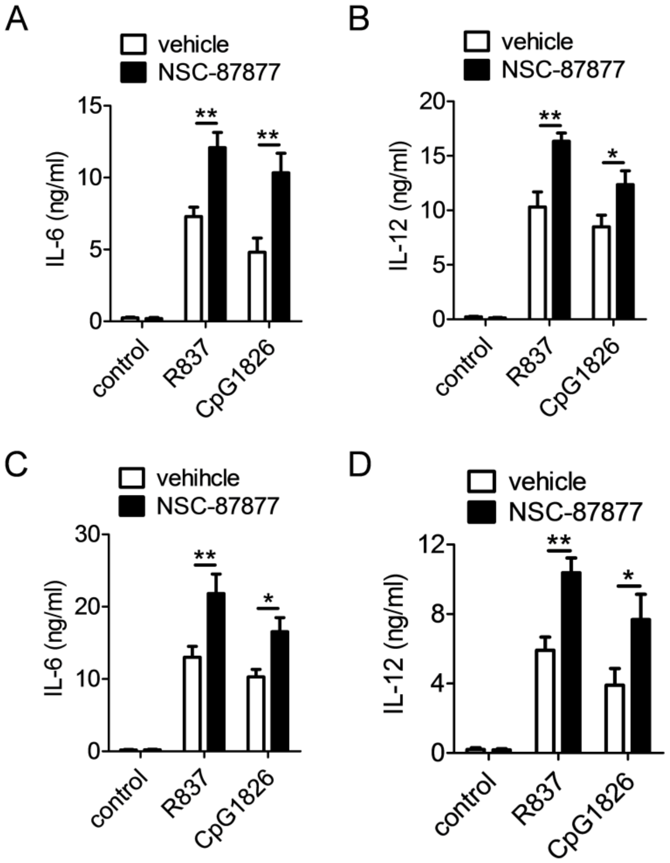

Figure S3 Effect of NSC-87877 on R837 and CpG 1826 induced expression of proinflammatory cytokines in BMDMs and BMDCs

Murine BMDMs and BMDCs were pretreated with NSC-87877 (1 μM) followed by stimulation of R837 (1 μg/ml) and CpG 1826 (0.5 μM) for 6 hours. **(A,B)** Q-PCR analysis of the expression of IL-6 **(A)** and IL-12 **(B)** in BMDMs. **(C,D)** Q-PCR analysis of the expression of IL-6 **(C)** and IL-12 (D) in BMDCs. The data shown represent the means of three independent experiments and the error bars represent the S.E.M. * $p < 0.05$, ** $p < 0.01$, as determined by ANOVA tests.

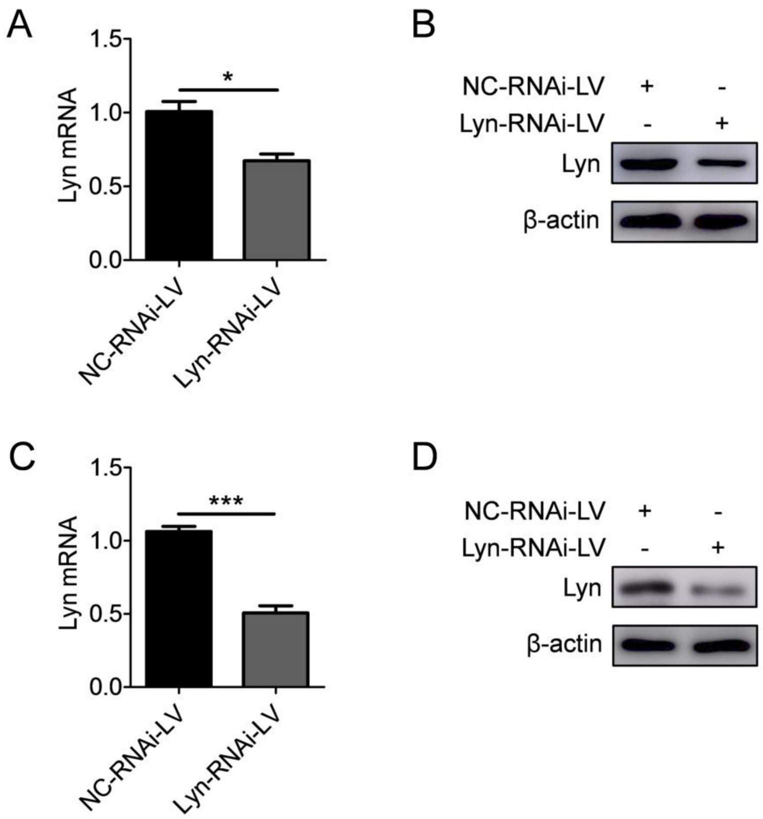

Figure S4 Knockdown effect of Lyn in BMDMs and BMDMs

(A) Q-PCR analysis of the expression of Lyn in BMDMs infected with lentivirus expressing negative control-RNAi (NC-RNAi-LV) or Lyn-specific RNAi (Lyn-RNAi-LV). **(B)** Western blot analysis of Lyn expression in BMDMs infected with NC-RNAi-LV or Lyn-RNAi-LV. **(C)** Q-PCR analysis of the expression of Lyn in BMDCs infected with lentivirus expressing negative control-RNAi (NC-RNAi-LV) or Lyn-specific RNAi (Lyn-RNAi-LV). **(D)** Western blot analysis of Lyn expression in BMDCs infected with NC-RNAi-LV or Lyn-RNAi-LV. The data shown represent the means of three independent experiments and the error bars represent the S.E.M. * $p < 0.05$, *** $p < 0.001$, as determined by *t*-test.

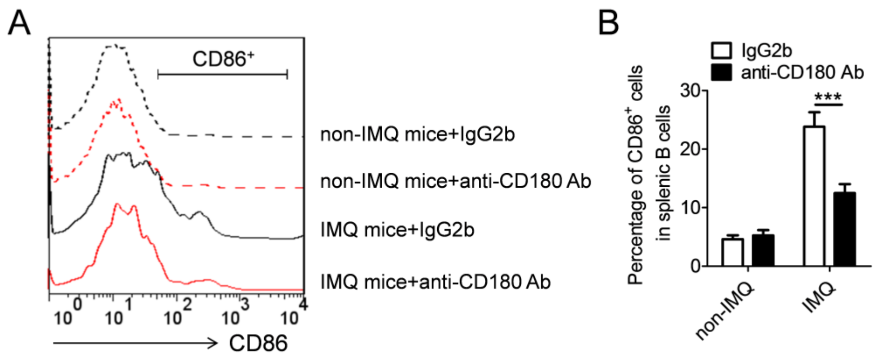

Figure S5 Effect of anti-CD180 Ab treatment on the activation of splenic B cells in IMQ-treated mice.

(A,B) Flow cytometric analysis of CD86 expression on splenic B cells in all groups of mice. The data are shown as the means ± SEM (n=7 mice/group). *** $p < 0.001$, as determined by ANOVA tests.

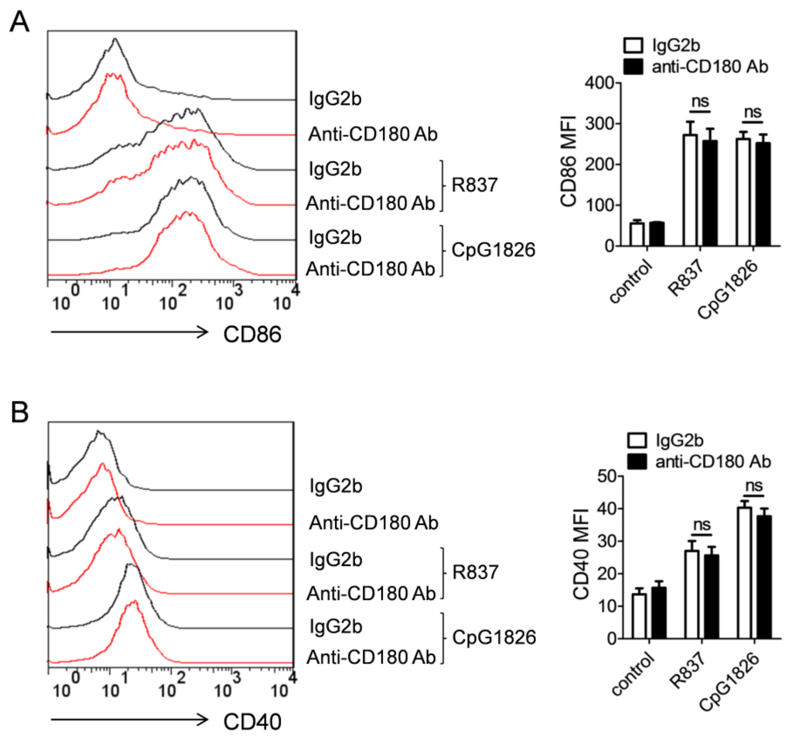

Figure S6 Effect of anti-CD180 Ab treatment on the activation of B cells *in vitro*.

Splenic B cells isolated from C57BL/6 mice were pretreated with anti-CD180 antibody (0.2 μg/ml) followed by stimulation of R837 (1 μg/ml) and CpG 1826 (0.5 μM). Flow cytometric analysis of CD86 **(A)** and CD40 **(B)** expressions at 24 hours. The data shown represent the means of three independent experiments and the error bars represent the S.E.M. ns denotes $p > 0.05$.

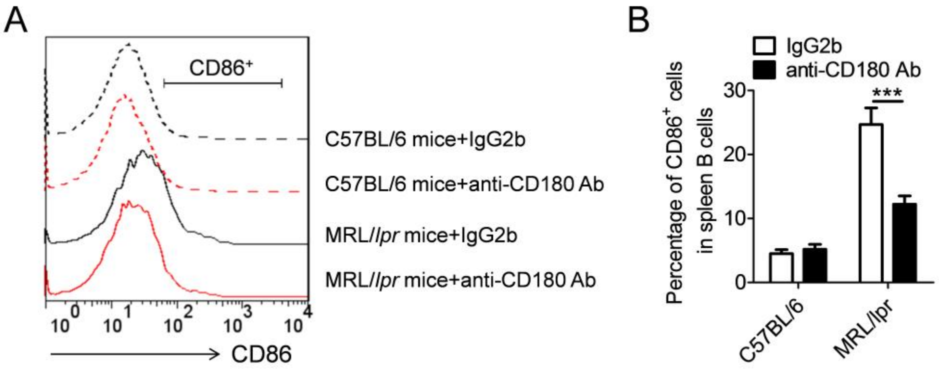

Figure S7 Effect of anti-CD180 Ab treatment on the activation of splenic B cells in MRL/*lpr* mice.

(A, B) Flow cytometric analysis of CD86 expression on splenic B cells in all groups of mice. The data are shown as the means ± SEM (n=7 mice/group). *** $p < 0.001$, as determined by ANOVA tests.
